# Randomised Clinical Trial to Evaluate the Efficacy of Acetazolamide for the Treatment of Cystoid Fluid Collections in X‐Linked Retinoschisis: The AXIS Trial

**DOI:** 10.1111/ceo.70146

**Published:** 2026-08-11

**Authors:** Jonathan Hensman, Hind Almushattat, Roselie M. H. Diederen, Tien‐En Tan, Peilun Dai, Coen de Vente, Daniel S. W. Ting, Maria M. van Genderen, Jeannette Ossewaarde‐van Norel, Redmer van Leeuwen, Martha J. H. Tjon‐Fo‐Sang, Clara I. Sánchez, M. Dominik Fischer, Maximilian Pfau, Jos W. R. Twisk, Camiel J. F. Boon

**Affiliations:** ^1^ Department of Ophthalmology Amsterdam University Medical Centre Amsterdam the Netherlands; ^2^ Nuffield Laboratory of Ophthalmology, Nuffield Department of Clinical Neurosciences University of Oxford Oxford UK; ^3^ Oxford Eye Hospital Oxford University Hospitals NHS Foundation Trust Oxford UK; ^4^ Singapore Eye Research Institute Singapore National Eye Centre Singapore Singapore; ^5^ Institute of High Performance Computing Agency for Science, Technology and Research (A*STAR) Singapore Singapore; ^6^ Quantitative Healthcare Analysis (qurAI) Group, Informatics Institute University of Amsterdam Amsterdam the Netherlands; ^7^ Amsterdam UMC Location University of Amsterdam Biomedical Engineering and Physics Amsterdam the Netherlands; ^8^ Department of Ophthalmology University Medical Centre Utrecht Utrecht the Netherlands; ^9^ Bartiméus Diagnostic Centre for Complex Visual Disorders Zeist the Netherlands; ^10^ The Rotterdam Eye Hospital and the Rotterdam Ophthalmic Institute Rotterdam the Netherlands; ^11^ Department of Ophthalmology University of Bonn Bonn Germany; ^12^ Department of Epidemiology and Data Science Amsterdam University Medical Centre Amsterdam the Netherlands; ^13^ Department of Ophthalmology Leiden University Medical Centre Leiden the Netherlands

**Keywords:** acetazolamide, carbonic anhydrase inhibitor, central subfield thickness, cystoid macular oedema, X‐linked juvenile retinoschisis

## Abstract

**Background:**

This trial aimed to evaluate the efficacy of oral acetazolamide in reducing cystoid fluid collections (CFC) and improving visual function in patients with X‐linked retinoschisis (XLRS).

**Methods:**

In this investigator‐initiated, single centre, open‐label, randomised controlled trial, XLRS patients aged ≥ 12 years with fovea‐involving CFC at baseline were eligible. Participants were randomised 1:1 to receive either oral acetazolamide (500 mg/day for 16 weeks, followed by tapering or discontinuation based on response) or no treatment (control) for 32 weeks. Seven study visits were completed over 32 weeks. The primary outcome was central subfield thickness (CST). Secondary outcomes included functional and structural measures, as well as patient‐reported visual disability.

**Results:**

A total of 19 patients were enrolled: 10 received acetazolamide (17 eyes), and nine were monitored without treatment (16 eyes). The difference in CST from baseline up to week 32 between the treatment and control groups was on average +2.7 μm (*p* = 0.9; 95% CI, −68.1 to 73.5 μm). Retinal sensitivity on microperimetry showed a modest statistically significant improvement favouring acetazolamide. However, no significant improvements were observed for best‐corrected visual acuity, low‐luminance visual acuity, cystoid fluid collection volume or patient‐reported visual disability in the treatment group compared to the control group.

**Conclusion:**

This study found no clinically meaningful benefit of oral acetazolamide for reducing CFC or improving visual function in the overall XLRS population. Although structural and functional outcomes were comparable between groups overall, a small subset of patients may experience benefit, suggesting a personalised treatment approach may be warranted.

**Abbreviations**
BCVA: best‐corrected visual acuity
CAI: carbonic anhydrase inhibition
CFC: cystoid fluid collections
CST: central subfield thickness
ETDRS: Early Treatment of Diabetic Retinopathy Study
IQR: interquartile range
LLVA: low luminance visual acuity
OCT: optical coherence tomography
RS1: retinoschisin‐1
SD: spectral‐domain
XLRS: X‐linked retinoschisis

## 1 Introduction

X‐linked retinoschisis (XLRS) is a monogenic inherited retinal disease affecting 1:5000 to 1:30000 males from early childhood with progressive bilateral central vision loss [1]. XLRS is caused by pathogenic variants in the *RS1* gene, which encodes retinoschisin, a protein essential for signal transduction and retinal integrity. A hallmark feature of XLRS is the presence of cystoid fluid collections (CFC) within the macula, which are detectable on spectral‐domain optical coherence tomography (SD‐OCT) in nearly all affected individuals [1, 2]. Although visual acuity in XLRS is often relatively stable during the first two decades of life, it is followed by a slow, progressive decline due to macular atrophy resulting from CFC coalescence and outer retinal thinning [1, 3, 4]. Consequently, therapeutic strategies aimed at reducing CFC have been proposed to preserve retinal architecture and visual function. RS1 gene therapy trials have yielded inconsistent results [5, 6], whilst an ongoing subretinal gene therapy trial (NCT05878860) reported encouraging preliminary findings.

Meanwhile, several retrospective and prospective studies have evaluated the use of topical and systemic formulations of carbonic anhydrase inhibitors (CAI) in XLRS, reporting variable effects on macular morphology and visual acuity. Whilst some studies demonstrated modest improvements in central retinal thickness or visual function [7, 8], others showed no clear benefit [3, 4, 9, 10, 11, 12].

However, most of these prior studies have been limited by their size, heterogeneous treatment regimens, and importantly, the absence of parallel untreated control groups. Previous work has shown that both CFC and visual function may fluctuate over time in XLRS as part of the natural disease course, raising concern that treatment effects may have been overestimated in uncontrolled designs [13, 14]. Although CAI efficacy for XLRS has been studied, an evidence‐based consensus on the optimal clinical management of CFC is still lacking due to the absence of prospective, randomised controlled trials. The AXIS trial was therefore designed as a randomised controlled study to evaluate the efficacy of oral acetazolamide in reducing CFC and improving visual function in patients with XLRS. The primary objective was to assess the effect of acetazolamide on central subfield thickness (CST) over a 32‐week period compared with observation alone. Secondary objectives included evaluation of best‐corrected visual acuity (BCVA), low‐luminance visual acuity (LLVA), retinal sensitivity assessed by microperimetry, CFC volume (CFCV) quantified using artificial intelligence–based segmentation and patient‐reported visual disability.

## 2 Methods

### 2.1 Study Design

The AXIS trial was an investigator‐initiated, single centre, open‐label, randomised controlled trial consisting of seven visits conducted at the Amsterdam University Medical Centre (Amsterdam, the Netherlands). This study adhered to the Declaration of Helsinki and was approved by the institutional ethics committee (NL80249.018.22). The trial was registered at ClinicalTrials.gov (identifier, NCT06114537). We obtained written informed consent from all enrolled patients prior to their participation. Male patients aged ≥ 12 years with a molecularly confirmed diagnosis of XLRS, or a clinical diagnosis consistent with XLRS based on characteristic imaging findings and inheritance pattern, were eligible. Inclusion required the presence of fovea‐involving CFC on SD‐OCT in at least one eye at baseline. Key exclusion criteria included BCVA worse than 20/200, ocular comorbidities affecting visual function, recent CAI use (< 3 months), contraindications to acetazolamide or concurrent use of interacting medications. The complete list of inclusion and exclusion criteria is found in Table 1. The detailed methods protocol is available in the Supporting Information Files.

**TABLE 1:** Inclusion and exclusion criteria of the AXIS trial.

Inclusion criteria ≥ 12 years of age and able to give written informed consent Confirmed diagnosis of XLRS Foveal CFC on SD‐OCT at the baseline examination
Exclusion criteria Presence of amblyopia or any other ocular diseases (e.g., glaucoma, perforating trauma) that could contribute to visual dysfunction, aside from XLRS BCVA of 20/200 or worse (Snellen equivalent) Concurrent treatment with CAI (topical or systemic), use of any CAI within 3 months before the expected start of the trial Intolerance or allergy to ocular anaesthetic eye drops (oxybuprocaine 0.4%) or mydriatics (tropicamide 0.5%, phenylephrine 5%) Contraindications to receiving acetazolamide Severe hepatic impairment Severe renal insufficiency Sodium or potassium depletion Addison's disease Hyperchloremic acidosis Cor pulmonale Chronic non‐congestive angle‐closure glaucoma Use of medications that interact with acetazolamide Folic acid antagonists (methotrexate, trimethoprim, pyrimethamine) Oral anticoagulants Hypoglycaemic agents (metformin) Aspirin Cardiac glycosides (digoxin) Diuretics (e.g., thiazides, loop diuretics) Anticonvulsants (phenytoin, primidone, carbamazepine) Carbonic anhydrase inhibitors (methazolamide, dorzolamide, brinzolamide, diclofenamide, ethoxzolamide and zonisamide) Procaine Sodium hydrogen carbonate Cyclosporine Ephedrine, methadone, amphetamine, quinidine, lithium

Abbreviations: BCVA = Best‐corrected visual acuity; CAI = carbonic anhydrase inhibition; CFC = cystoid fluid collections; OCT = optical coherence tomography; SD = spectral‐domain; XLRS = X‐linked retinoschisis.

### 2.2 Randomisation

Patients were randomised 1:1 to receive acetazolamide or observation alone using a web‐based random number generator with block randomisation using block sizes of 5. Due to the absence of placebo treatment, masking of participants and investigators was not feasible.

### 2.3 Procedures

Participants attended seven study visits over 32 weeks (baseline; weeks 2, 4, 12, 16, 20 and 32). Visits were scheduled at consistent times of day to minimise diurnal variation in retinal morphology and visual function [14]. At each visit, BCVA and LLVA were assessed using Early Treatment of Diabetic Retinopathy Study (ETDRS) charts, retinal sensitivity was measured using Macular Integrity Assessment microperimeter (MAIA; iCare, Padova, Italy) and OCT imaging of the macula was performed. Patient‐reported visual disability was assessed at baseline, weeks 12 and 16 and week 32 using the Dutch version of the Michigan Retinal Degeneration Questionnaire (MRDQ) [15]. Fundus photography and fundus autofluorescence imaging were obtained at baseline and at the final visit. Multimodal imaging consisted of ultra‐widefield retinal images of the fundus (200° image) acquired on the Optos California device (Optos PLC, Dunfermline, United Kingdom), and SD‐OCT and FAF imaging acquired on the Heidelberg Spectralis HRA + OCT device (Heidelberg Engineering Inc., Heidelberg, Germany).

### 2.4 Acetazolamide Treatment

Patients assigned to treatment initiated oral acetazolamide at 500 mg/day (250 mg bid) for 16 weeks. At week 16, treatment response was assessed based on change in central foveal thickness (CFT). A reduction of ≥ 25% from baseline was considered clinically meaningful [7, 9]. Responders continued treatment at a reduced dose (125 mg bid), whilst non‐responders discontinued acetazolamide and entered observation. Responders who later showed relapse at week 20, defined as loss of ≥ 25% CFT reduction, were switched back to the full dose (250 mg twice daily) until the final visit (Figure 1). Dose adjustments were permitted for tolerability. Treatment adherence was monitored by tablet counts, and adverse events were recorded at each visit.

**FIGURE 1:**
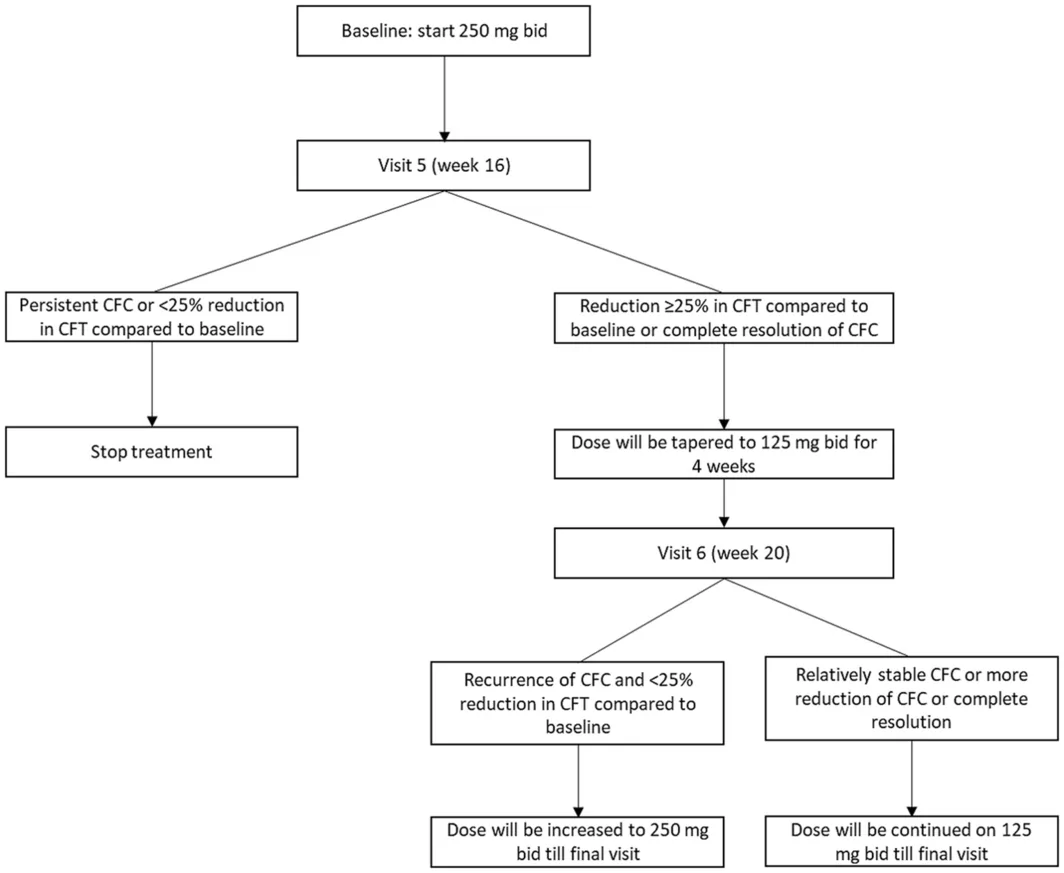
Flowchart of acetazolamide treatment protocol. CFC = cystoid fluid collections; CFT = central foveal thickness.

### 2.5 Outcomes

The primary outcome was the difference in CST on SD‐OCT over the 32‐week study period on average over time between the treatment and control groups. Previous XLRS studies evaluating carbonic anhydrase inhibitors have primarily reported treatment effects using CST or CFT as the main structural outcome measures. In line with the existing XLRS literature, a 25% reduction in CST was prespecified as clinically meaningful and was used for the sample size calculation. Accordingly, the trial was powered to detect a 25% reduction in CST at week 32. Secondary outcomes included differences in BCVA, LLVA, retinal sensitivity on microperimetry, CFCV and MRDQ scores. Adverse events were monitored throughout the study.

### 2.6 Imaging and Functional Analyses

CST was derived from automated retinal layer segmentation by the Spectralis in‐built software with manual correction when necessary. CFCV was quantified using a validated artificial intelligence–based segmentation model (details provided in Supporting Informations) [16]. Retinal sensitivity was analysed using smoothed and interpolated sensitivity maps to derive unbiased estimates of sensitivity across the tested area, including analyses restricted to baseline CFC regions (see Supporting Informations and Figure 2) [17, 18]. Microperimetry testing was considered reliable if fixation loss was ≤ 30% and the BCEA95 < 50 deg^2^ [19, 20]. MRDQ responses were scored using item response theory to generate domain‐specific theta scores [21].

**FIGURE 2:**
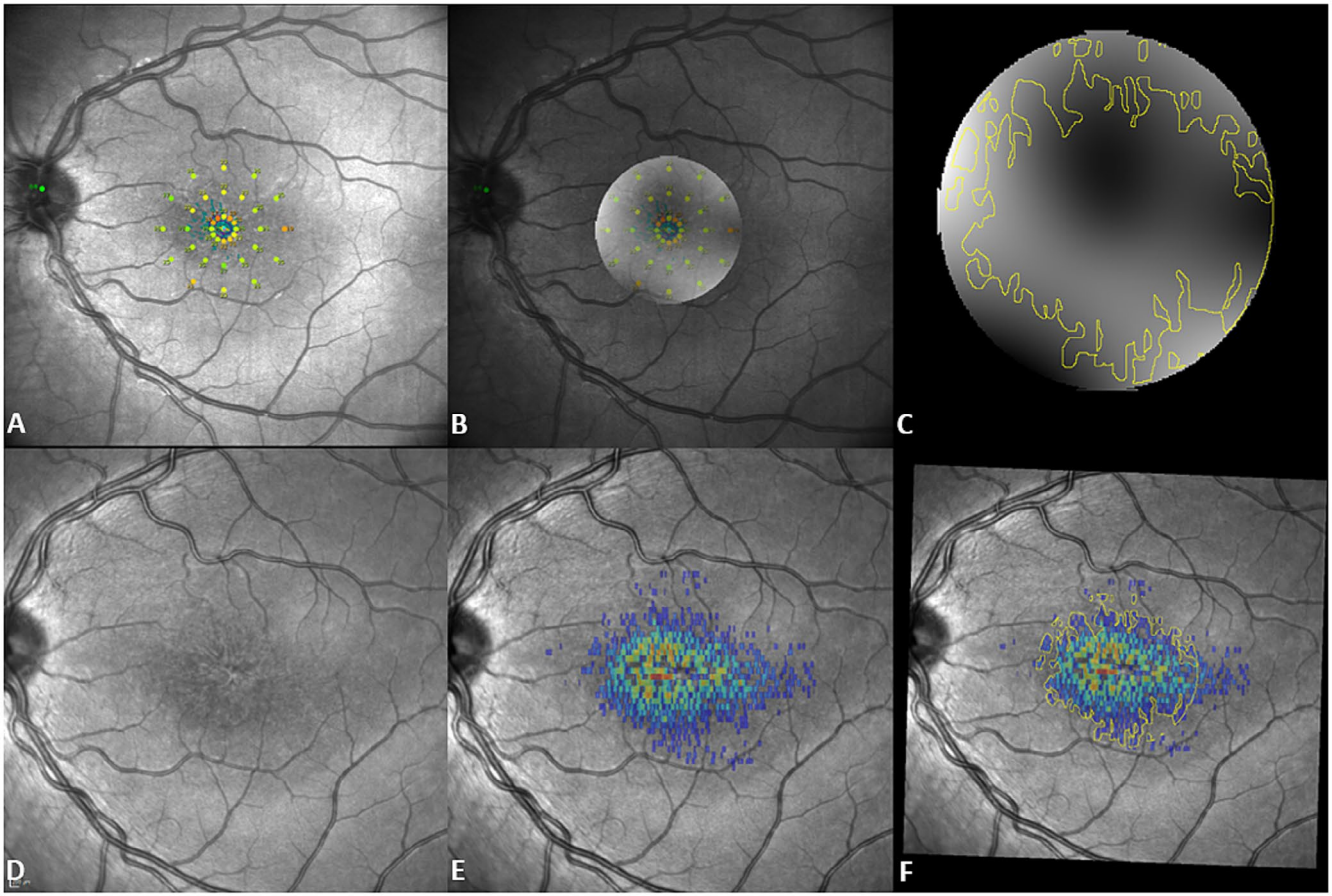
Steps required to measure retinal sensitivity on microperimetry when the tested area is restricted to area containing cystoid fluid collections (CFC). (A) An example of the standard Point threshold values overlaid onto a fundus image output given by the MAIA microperimeter. (B) Two dimensional smoothed, interpolated heatmap generated using thin plate spline. (D) An example of the standard Spectralis scanning laser ophthalmoscopy (SLO) image of the same patient. (E) Heatmap of CFC predictions overlaid onto the Spectralis SLO‐image. (F) Manually aligned and transformed the SLO‐image to the interpolated sensitivity map by identifying anatomical landmarks. (C) Two dimensional smoothed, interpolated heatmap of the point sensitivities restricted to CFC‐area.

### 2.7 Statistical Analyses

A target sample size of 18 patients was calculated to detect a clinically relevant treatment effect, defined as a 25% reduction in CST at week 32, assuming a standard deviation of 90 μm, with 80% power at a two‐sided *α* level of 0.05 and allowing for a 10% dropout rate. This threshold was based on prior studies in XLRS that considered a ≥ 22.4% reduction in CST to be clinically relevant [7, 9]. The calculation was originally based on one eye per participant. However, both eyes were included in the final analysis when eligible, which increased the effective sample size. To account for within‐subject and between‐eye correlation, a correlation coefficient of 0.6 was assumed. Each participant underwent seven visits over a 32‐week period (baseline visit plus six follow‐up visits). All analyses followed the intention‐to‐treat principle. Continuous variables are presented as mean ± standard deviation (SD) or median with interquartile range (IQR), depending on distribution. Categorical variables are reported as frequencies and percentages. Mixed‐effects linear regression models were used to evaluate treatment effect for continuous longitudinal outcomes. For outcomes assessed at baseline and follow‐up visits, baseline measurements were included as covariates. Two longitudinal analyses were performed. The first included only treatment, which resulted in a treatment effect on average over time, while the second included treatment, time and the time‐by‐treatment interaction, which resulted in treatment effects at each follow‐up visit. Furthermore, all analyses were adjusted for laterality (eye) and a random intercept on patient level was added to the model to account for the intra‐subject correlation. Associations between changes in structural (CST and CFCV) and functional outcomes (BCVA, LLVA and retinal sensitivity (restricted to CFC area)) were also analysed using mixed‐effects linear regression models. These models included the changes between subsequent measurements and the analyses were adjusted for treatment group and laterality (eye), and included a random intercept on patient level to account for the intra‐subject correlation.

All statistical analyses were conducted using SPSS Statistics (version 25.0; IBM Corp., Armonk, New York, USA), except for the calculations of the θ‐scores for each MRDQ domain using R software version 4.4.3 (R Foundation for Statistical Computing, Vienna, Austria) and the mixed‐effects linear regression models, which were performed in Stata (version 15.1; StataCorp, College Station, Texas, USA).

## 3 Results

In total, 142 XLRS patients were screened for eligibility. Of these, 65 met the inclusion criteria and were approached for participation; 26 consented to enrol. Seven participants were subsequently deemed ineligible due to the absence of CFC on SD‐OCT at the baseline visit (*n* = 4), use of medications contraindicated with acetazolamide (*n* = 2) and withdrawal of consent due to unwillingness to initiate treatment within a randomised trial (*n* = 1). Between 20 January 2023, and 2 February 2024, 19 patients were randomised to either acetazolamide treatment (*n* = 10) or observation without treatment (*n* = 9; Figure 3). In the treatment group, three eyes from three patients were excluded due to the absence of foveal CFC at baseline (*n* = 2) and BCVA < 20/200 (*n* = 1). In the control group, two eyes from two patients were excluded due to macular pucker (*n* = 1) and absence of foveal CFC at baseline (*n* = 1). All enrolled patients completed the study. All patients attended all follow‐up visits, except for one participant in the treatment group who missed a follow‐up visit at week 20. The median treatment compliance was 95.6% (IQR, 44.9%–98.5%), with this value skewed by one patient who demonstrated a markedly lower compliance rate of 29.4%. Two protocol deviations occurred: one patient in the treatment group discontinued medication after visit 4 (week 12) due to poor adherence, and another patient in the treatment group attended a follow‐up visit outside the predefined visit window due to a self‐limiting vitreous haemorrhage unrelated to the study drug.

**FIGURE 3:**
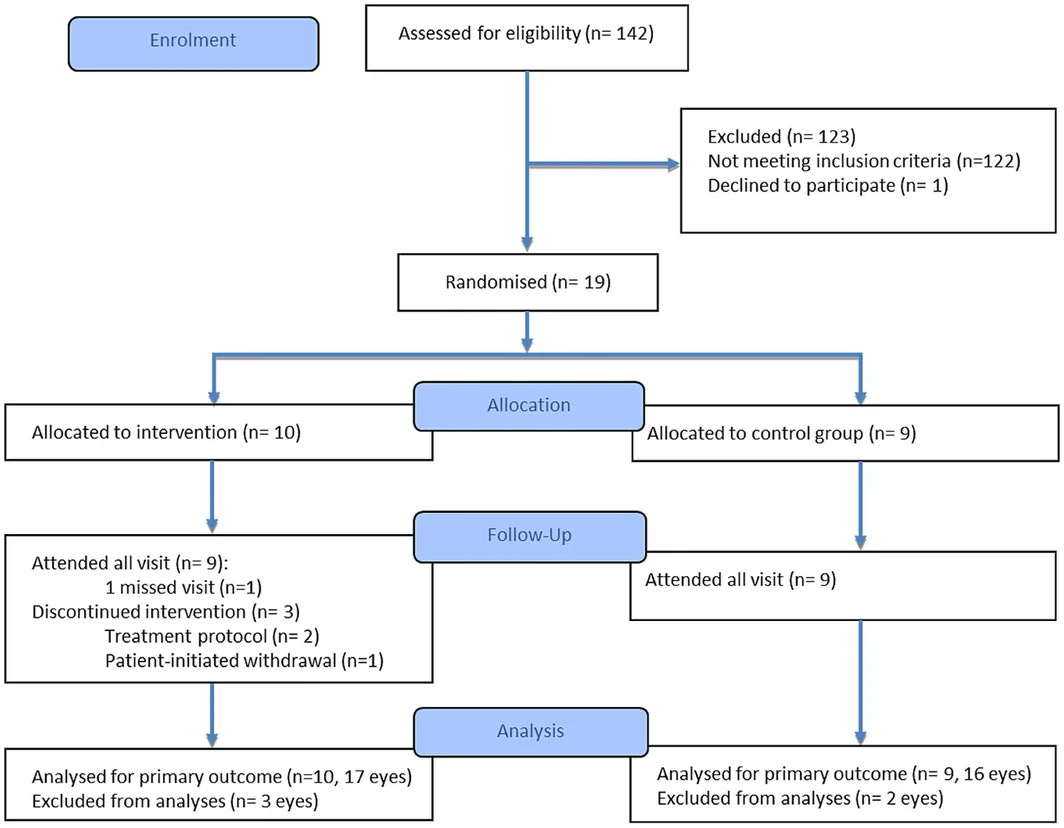
Flow diagram showing the trial profile for the study population.

Baseline demographics and clinical characteristics were comparable between the two groups (Table 2). Genetic testing data were available for 5 of 9 patients in the control group and for 8 of 10 patients in the treatment group. Patients without genetic confirmation had a clinically characteristic XLRS phenotype with compatible inheritance patterns, and the absence of genotyping reflected historical clinical practice rather than negative genetic testing results. The most common pathogenic variant was the Dutch founder *RS1* variant c.214G>A; p.Glu72Lys, which was observed in 5 of 19 patients (see Table S1).

**TABLE 2:** Baseline clinical characteristics of X‐linked retinoschisis patients.

Subject‐level characteristics	*N* = 10	*N* = 9
Age (years), median (IQR)	21.8 (17.9–29.4)	25.7 (20.2–43.2)
BCVA (ETDRS letters), mean ± SD	60.0 ± 11.2	67.2 ± 10.6
Disability score per domain (*θ*), mean ± SD		
Central vision	−0.2 ± 0.5	−0.1 ± 0.5
Colour	−0.6 ± 0.6	−0.5 ± 0.7
Contrast sensitivity	−0.5 ± 0.6	−0.7 ± 0.6
Scotopic function	−0.7 ± 0.6	−1.1 ± 0.6
Photopic peripheral function	−0.8 ± 0.6	−1.0 ± 0.5
Mesopic peripheral function	−0.7 ± 0.5	−1.1 ± 0.6
Photosensitivity	−0.6 ± 0.4	−1.0 ± 0.6

Eye‐level characteristics	*N* = 17	*N* = 16
LLVA (ETDRS letters), mean ± SD	50.2 ± 10.7	52.8 ± 11.6
Retinal sensitivity on MP (dB), mean ± SD^a^	19.2 ± 3.3	18.8 ± 4.0
Retinal sensitivity on MP restricted to fluid area (dB), mean ± SD^a^	19.1 ± 3.2	18.4 ± 4.5
CST (μm), mean ± SD	468.8 ± 99.4	426.1 ± 128.6
CFCV (mm^3^), mean ± SD^b^	1.1 ± 0.8	0.7 ± 0.5

Abbreviations: BCVA = best‐corrected visual acuity; CFCV = cystoid fluid collections volume; CST = central subfield thickness; ETDRS = Early Treatment of Diabetic Retinopathy Study; IQR = interquartile range; LLVA = low luminance visual acuity.

^a^ Microperimetry data available for 14 eyes in the control group due to unreliable fixation at baseline for two eyes.

^b^ CFCV data available for 15 eyes in the control group due to quality exclusions at baseline for one eye.

Amongst treated patients, three met the predefined ≥ 25% CFT reduction threshold at week 16 and continued at 125 mg bid until study completion, two required re‐escalation to 250 mg bid from week 20 due to loss of response, and three discontinued treatment for insufficient response. One patient requested a reduced dose 6 days after initiating treatment due to adverse events (nausea, polyuria and dry mouth) but subsequently demonstrated sustained CFT reduction throughout follow‐up. One patient discontinued treatment after week 12 because of poor adherence, unrelated to adverse events.

### 3.1 Primary Endpoint

Longitudinal analysis revealed no significant difference in CST on average over time between the two groups: +2.7 μm (*p* = 0.9; 95% CI, −68.1 to 73.5). Figures 4 and 5 illustrate fluctuations in CFC over time in the control group, as well as heterogeneous effects of acetazolamide on CFC amongst patients in the treatment group. Notably, the largest treatment effect of acetazolamide on CST occurred at week 4, which was, however, not significant (Table S2). Complete resolution of CFC was observed in 2 of 17 eyes (1 of 10 patients) in the acetazolamide group and in 2 of 16 eyes (2 of 9 patients) in the control group.

**FIGURE 4:**
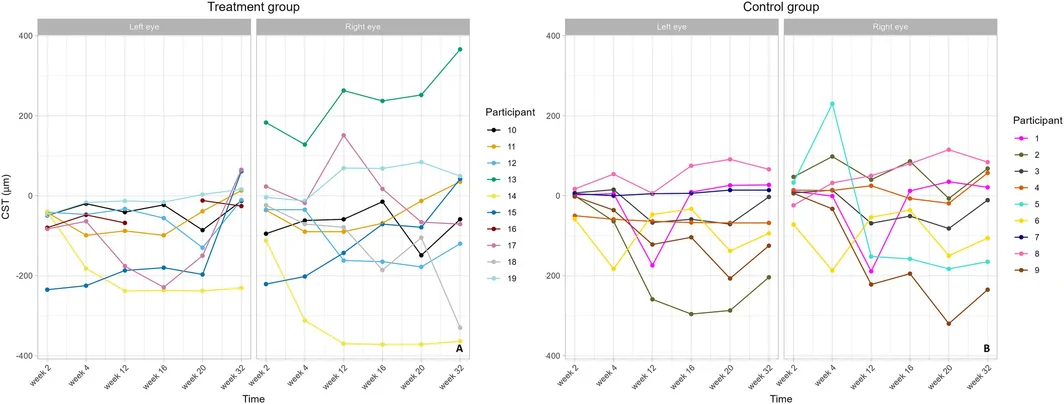
Changes in central subfield thickness (CST, in μm), from baseline to follow‐up visits (week 2, 4, 12, 16, 20 and 32) in the control and treatment groups. Individual eye trajectories are shown separately for left and right eyes. (A) Control group. (B) Treatment group.

**FIGURE 5:**
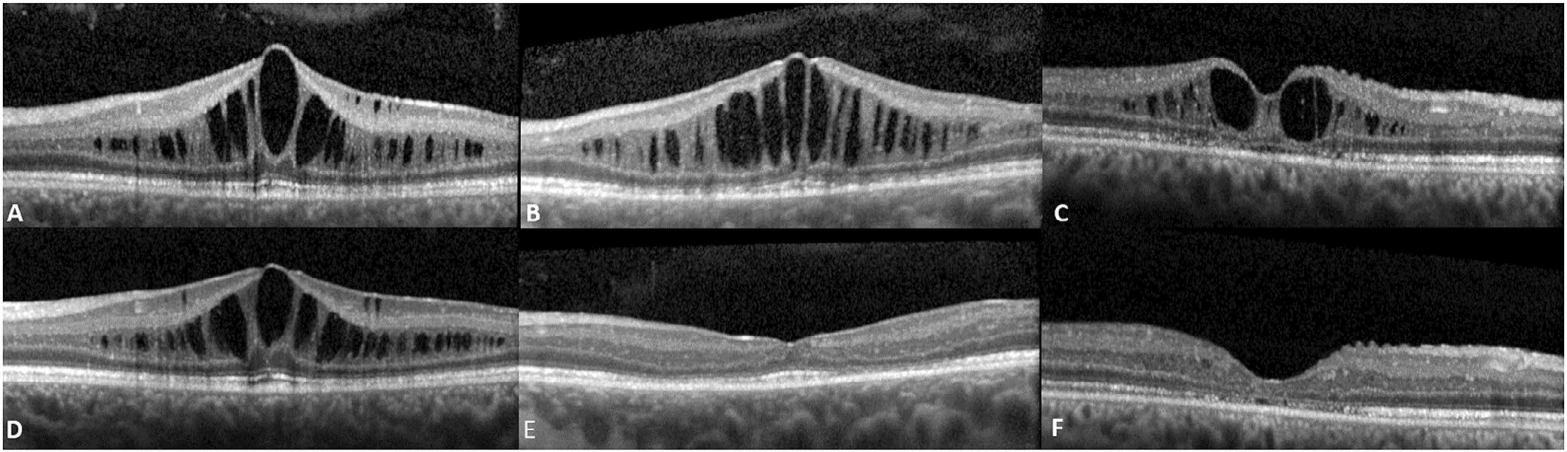
Spectral‐domain OCT images through the fovea of two eyes from two X‐linked retinoschisis patients treated with acetazolamide (A, B) and one eye from a control patient (C) at baseline (top row). The corresponding follow‐up scans at week 20 are shown in the bottom row. Acetazolamide had a modest effect on cystoid fluid collections (CFC) in one treated patient (D), whilst it resulted in complete CFC resolution in the other treated patient (E). The control eye also showed complete resolution of CFC at week 20 (F).

### 3.2 Secondary Endpoints

On average over time, the difference in BCVA between groups was +0.9 ETDRS letters (*p* = 0.4; 95% CI, −1.6 to 3.0). The longitudinal analysis indicated no significant effect of treatment duration on BCVA at the group level (see Table S3). However, at the individual level, 3 out of 17 eyes (17.6%) in the treatment group showed an improvement of ≥ 10 ETDRS letters at any visit after baseline, compared with none in the control group. Only one eye in the treatment group demonstrated a sustained ≥ 10‐letter improvement across all visits (Figure 6). Moreover, longitudinal analysis revealed no significant difference in LLVA on average over time between the two groups: +1.9 ETDRS letters (*p* = 0.2; 95% CI, −1.2 to 5.0). Improvements of ≥ 10 ETDRS letters in LLVA occurred in 8 of 17 treated eyes (47.1%) and 5 of 16 control eyes (31.3%) at any follow‐up visit (Figure S1). Of all 230 recorded examinations of retinal sensitivity on microperimetry in all included eyes, 15 (6.5%) were excluded for the following reasons: fixation loss > 30% (*n* = 2), too many false negatives (*n* = 2), corrupt saved file (*n* = 1), fixation deviation from other visit examinations (*n* = 10). The median total tested areas and tested areas restricted to CFC‐area on microperimetry at the baseline visit were 8.2 mm^2^ (IQR 6.8–8.5) and 7.1 mm^2^ (IQR 6.1–8.1) in the treatment group, and 7.8 mm^2^ (IQR 6.5–8.6) and 6.6 mm^2^ (IQR 3.1–7.4) in the control group. Statistically significant between‐group differences in retinal sensitivity on MP on average over time, favouring acetazolamide, were observed for both the total tested area (+1.3 dB; *p* = 0.03; 95% CI, 0.1 to 2.6) and the CFC‐restricted region (+1.3 dB; *p* = 0.03; 95% CI, 0.1 to 2.5). The change in retinal sensitivity on MP over time at the individual level is depicted in Figures S2 and S3.

**FIGURE 6:**
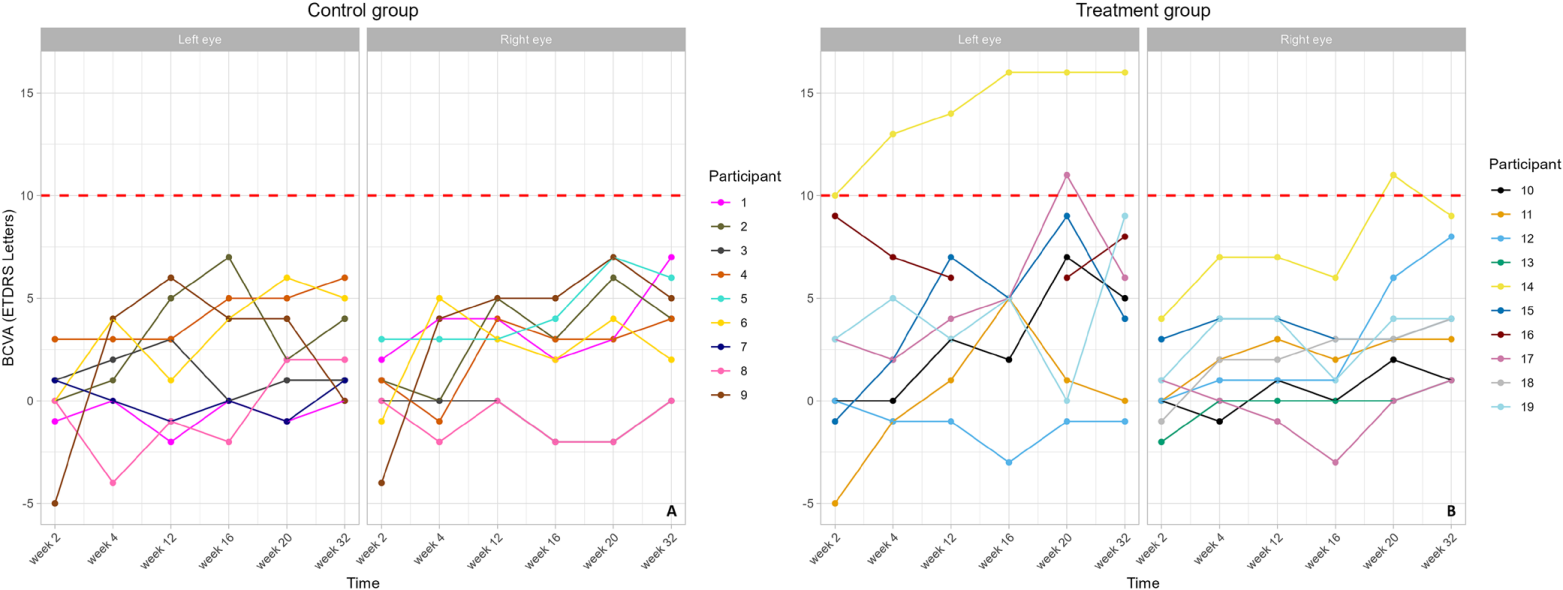
Changes in best‐corrected visual acuity (BCVA), measured in Early Treatment Diabetic Retinopathy Study (ETDRS) letters, from baseline to follow‐up visits (week 2, 4, 12, 16, 20 and 32) in the control and treatment groups. Individual eye trajectories are shown separately for left and right eyes. The red dashed line represents a +10 ETDRS letter gain, denoting the threshold for clinically meaningful improvement. (A) Control group. (B) Treatment group.

The CFCV was calculated for the total of 230 SD‐OCT scans of the included 33 eyes of 19 patients. Of these, 9 CFCV calculations (3.9%) were excluded from analyses due to poor segmentation accuracy after the quality check. No significant between‐group difference in CFCV on average over time was observed: +0.01 mm^3^ (*p* = 0.9; 95% CI, −0.2 to 0.2). The change of CFCV over time at the individual level is depicted in Figure S4.

In addition to the structural and functional endpoints, this study investigated the patient‐reported visual disability in our trial cohort on the MRDQ. Table 2 summarises the disability score for each of the seven domains at baseline in the two groups. The median *θ* ranged from −0.98 to −0.21 in the treatment group and from −1.26 to −0.13 in the control group. The patients experienced most disability with central vision and contrast sensitivity in the treatment group, and central vision and colour vision in the control group. Over the entire study period, acetazolamide treatment did not significantly reduce perceived disability in any visual domain (see Tables S8–S9). The effect of acetazolamide on the structural and functional endpoints at each visit is summarised in the Tables S2–S7 and S9.

### 3.3 Structure–Function Associations

Associations between changes in structural parameters (CST and CFCV) and functional outcomes (BCVA, LLVA and retinal sensitivity on MP, both total and restricted to the CFC area) showed that a decrease in CST was significantly associated with an increase in visual function, that is, a 100 μm decrease in CST was associated with an increase of 0.9 ETDRS letters in BCVA (*p* < 0.001), a 1.21‐letter increase in LLVA (*p* < 0.001), a 0.5 dB increase in total MP sensitivity (*p* < 0.001) and a 0.6 dB increase in MP sensitivity restricted to the CFC area (*p* < 0.001). Similarly, a 1 mm^3^ decrease in CFCV was associated with a 2.4‐letter increase in BCVA (*p* < 0.001), a 3.4‐letter increase in LLVA (*p* < 0.001), a 1.3 dB increase in total MP sensitivity (*p* < 0.001) and a 1.5 dB increase in MP sensitivity over the CFC region (*p* < 0.001). These structure–function associations are summarised in Table S10.

### 3.4 Adverse Events

No serious adverse events related to acetazolamide were observed. Results for all adverse events are recorded in Table 3. One patient experienced a vitreous haemorrhage unrelated to the study drug. The most frequently reported drug‐related adverse events included paraesthesia in the hands or feet (7 patients; 22 occurrences), dysgeusia (6 patients; 11 occurrences) and polyuria (4 patients; 10 occurrences). One participant required dose reduction due to tolerability. No participants discontinued treatment due to adverse events.

**TABLE 3:** Adverse events reported between the baseline and final visit.

	Treatment group (*n* = 10)	
	All events	
	Number of patients (%)	Number of events
Serious adverse events	0 (0%)	0
Any event (anticipated or unanticipated)		
Vitreous haemorrhage	1 (10%)	1
Systemic treatment related adverse events		
Paraesthesia in the hand or leg	7 (70%)	22
Dysgeusia	6 (60%)	11
Polyuria	4 (40%)	10
Dry mouth	4 (40%)	7
Fatigue	5 (50%)	5
Nausea	4 (40%)	5
Dizziness	2 (20%)	4
Gastroesophageal reflux disease	1 (10%)	2
Tinnitus	1 (10%)	2
Dyspnoea	1 (10%)	1
Rash	1 (10%)	1
Diarrhoea	0 (0%)	0
Drowsiness	0 (0%)	0
Mood change (low mood)	0 (0%)	0

### 3.5 Other Findings

The most common findings on the fundus images were golden sheen seen in 11 patients (17 eyes), macular schisis in 8 patients (14 eyes) and peripheral retinoschisis in 8 patients (10 eyes). The FAF findings were normal for 2 out of 33 eyes (6.1%), whilst a characteristic spoke wheel pattern was seen in 28 eyes (84.8%). No changes were observed at the last visit compared to the baseline visit, except for the presence of retinoschisis and spoke wheel pattern on fundus imaging and/or FAF (Tables S11 and S12).

## 4 Discussion

This investigator‐initiated, open‐label, randomised controlled trial evaluated the efficacy of oral acetazolamide on CFC and visual function in XLRS patients. The results for the primary outcome of this AXIS trial did not meet the predefined threshold of a ≥ 25% reduction in CST, which was the target difference used for power calculations. Moreover, this trial demonstrated no significant and clinically relevant effect of acetazolamide in improving BCVA in the treated group compared to the control group. Until now, retrospective and prospective studies have shown inconsistent treatment effects of acetazolamide on BCVA and CST, with some studies showing a significant treatment effect on BCVA and/or CST [7, 8], whilst other studies have shown modest or no treatment effects [3, 4, 9, 10, 11, 12]. Most of these studies were retrospective and had different treatment protocols and follow‐up time, but most importantly lacked a proper control group. A notable exception is the prospective, randomised crossover trial by Gurbaxani et al. In their cohort of 11 patients, they found a statistically significant improvement in BCVA during the active treatment phase, but found no significant reduction in CST at the group level [11]. In contrast, the current AXIS trial included a larger cohort of 19 patients and employed a parallel‐group design. The inclusion of a sustained, untreated control arm allowed us to observe that CFC and BCVA fluctuate spontaneously over time as part of the natural disease course, as shown previously by Mautone et al. and Abalem et al., and visualised in our control group (Figures 4, 5, 6) [13, 14]. This parallel‐arm observation provides a key methodological lesson that functional gains reported in crossover or uncontrolled studies may partly reflect natural disease variability rather than a consistent pharmacological effect. Despite these design differences, both prospective trials ultimately reached convergent structural conclusions, with neither demonstrating a significant reduction in CST at the group level. Taken together, these findings indicate that acetazolamide does not provide a reliable, uniform benefit in the broader XLRS population and that treatment efficacy may have been overestimated in prior studies.

In our study, CFCV was included as a secondary outcome as a more accurate quantification of the CFC instead of using only CST. Similarly, CFCV showed no statistically significant treatment‐related change across visits. Consistent with findings by Testa et al., our study demonstrated a statistically significant improvement in retinal sensitivity across the full MP test area (+1.3 ± 0.6 dB), as well as within the region corresponding to CFC (+1.3 ± 0.6 dB) [7]. However, the magnitude of this improvement appears modest when considering the natural test–retest variability observed in inherited retinal diseases. This variability is quantified by the coefficient of repeatability, a statistical threshold that defines the smallest measurable change likely to reflect a true clinical difference, rather than random measurement noise. In other words, changes smaller than the coefficient of repeatability are likely attributable to natural fluctuations or device variability rather than to a treatment effect.

The 95% repeatability coefficient for mean sensitivity is likely to be in the range of about ±2.0 to ±2.5 dB [22, 23]. Based on these reference values, the changes observed in our study likely fall within the expected range of test–retest variability and may not represent clinically meaningful improvements.

Moreover, LLVA appeared more responsive at the individual level than standard BCVA, with a higher proportion of eyes demonstrating transient improvements. LLVA has been proposed as a more sensitive measure of macular dysfunction under low/dim light conditions compared to BCVA and may better reflect broader macular integrity beyond the fovea [24, 25].

Thus far, limited data were available on the patient‐perceived visual disability and subjective visual function in XLRS patients [12]. Our study provides valuable insight into the functional impact of XLRS from the patient's perspective. At baseline, the included patients reported the greatest disability in domains closely related to macular integrity, particularly central vision, colour vision and contrast sensitivity—findings consistent with the characteristic foveal schisis and macular dysfunction seen in XLRS. Acetazolamide treatment showed no statistically significant or clinically meaningful improvement across any of the MRDQ domains throughout the study period, which highlights the limitations in altering perceived visual function in a relatively stable XLRS cohort. Acetazolamide was generally tolerated, but adverse effects were common and contributed to treatment burden. Whilst no participants discontinued treatment due to adverse events, none elected to continue acetazolamide after study completion, citing limited perceived benefit relative to side effects. This highlights the importance of balancing efficacy with tolerability, particularly for long‐term management of relatively stable inherited retinal diseases.

Despite the lack of overall efficacy, one treated participant demonstrated sustained and meaningful improvements in both structural and functional outcomes across follow‐up. This finding suggests that a subset of patients may benefit from acetazolamide. We hypothesise that an intact central outer retina—particularly preservation of the ellipsoid zone and retinal pigment epithelium—may be essential for a therapeutic response. Future studies should investigate whether the structural integrity of the outer retina modulates treatment efficacy in XLRS. The relatively young age of patients within our cohort (with median ages of 21.8 years in the treatment group and 25.7 years in the control group) should also be considered when interpreting the limited functional changes observed during follow‐up. Longitudinal natural history studies have demonstrated that XLRS follows a prolonged plateau phase during the first three to four decades of life, during which BCVA often remains relatively stable with only minimal annual decline [1, 12, 26, 27]. This relative stability provides a relatively controlled baseline for interventional studies, as treatment‐related improvements are less likely to be confounded by rapid underlying disease progression [1, 26]. In contrast, structural fluctuations in cystoid fluid collections may still occur during this disease phase. Associations between structural and functional parameters reinforce the clinical relevance of anatomical changes. Reduction in CST and CFCV were significantly associated with improvements in BCVA, LLVA and MP‐sensitivity, independent of treatment allocation. These findings suggest that, although acetazolamide may not provide consistent or long‐term benefit, reducing CFC itself may remain a valid therapeutic target in XLRS and may enhance the efficacy of future gene therapy by addressing macular CFC prior to treatment.

This study has several strengths, including its randomised design and the incorporation of both established and novel functional outcome measures, like the retinal sensitivity on MP restricted to CFC area and the CFCV. All enrolled patients contributed to the primary analysis, with only one patient missing a single follow‐up visit. The trial's statistical power was enhanced beyond initial estimates through the inclusion of both eyes when eligible, with appropriate adjustment for inter‐eye correlation. However, there are notable limitations. The small sample size, relatively short follow‐up period and relatively young patients in our study may limit the generalisability of the findings to the broader XLRS population.

In conclusion, in the AXIS trial, we demonstrated that oral acetazolamide did not significantly improve CST, BCVA, LLVA, CFCV and patient‐reported visual disability on the MRDQ, compared with the control group, after adjusting for the natural fluctuations in visual function and CFC in XLRS. Whilst an increase in retinal sensitivity on microperimetry was observed in some patients in the treatment group, this improvement was transient and probably not clinically relevant. These findings suggest that acetazolamide does not provide meaningful functional or structural benefit on average in a broader XLRS population. However, our data indicate that subgroups of patients do respond, and in these responders, the strong structure–function correlations suggest that targeting CFC reduction may be worthwhile. Future research should explore whether specific anatomical or functional biomarkers, such as intact outer retinal layers, may help identify subgroups more likely to benefit from carbonic anhydrase inhibition.

## Funding

This research was supported by the Stichting Amsterdam UMC Foundation.

## Conflicts of Interest

The authors declare no conflicts of interest.

## Supporting information


**Figure S1:** Changes in low luminance visual acuity (LLVA), measured in Early Treatment Diabetic Retinopathy Study (ETDRS) letters, from baseline to follow‐up visits (week 2, 4, 12, 16, 20 and 32) in the control and treatment groups. Individual eye trajectories are shown separately for left and right eyes. The red dashed line represents a +10 ETDRS letter gain, denoting the threshold for clinically meaningful improvement. A, control group. B, treatment group.
**Figure S2:** Changes in retinal sensitivity on microperimetry (MP, in dB) from baseline to follow‐up visits (week 2, 4, 12, 16, 20 and 32) in the control and treatment groups. Individual eye trajectories are shown separately for left and right eyes. A, control group. B, treatment group.
**Figure S3:** Changes in retinal sensitivity on microperimetry (MP, in dB) with the tested area restricted to cystoid fluid collections (CFC) area from baseline to follow‐up visits (week 2, 4, 12, 16, 20 and 32) in the control and treatment groups. Individual eye trajectories are shown separately for left and right eyes. A, control group. B, treatment group.
**Figure S4:** Changes in cystoid fluid collections volume (CFCV, in mm^3^) from baseline to follow‐up visits (week 2, 4, 12, 16, 20 and 32) in the control and treatment groups. Individual eye trajectories are shown separately for left and right eyes. A, control group. B, treatment group.

**Data S1:** Supplementary Methods protocol.

**Table S1:** Genetic information on the pathogenic *Retinoschisin‐1* variant in the 19 subjects with X‐linked retinoschisis.
**Table S2:** Effect of acetazolamide on central subfield thickness across every visit.
**Table S3:** Effect of acetazolamide on best‐corrected visual acuity across every visit.
**Table S4:** Effect of acetazolamide on low luminance visual acuity across every visit.
**Table S5:** Effect of acetazolamide on retinal sensitivity on total tested area on microperimetry across every visit.
**Table S6:** Effect of acetazolamide on retinal sensitivity on tested area on microperimetry restricted to cystoid fluid collections‐area across every visit.
**Table S7:** Effect of acetazolamide on cystoid fluid collections volume across every visit.
**Table S8:** Effect of acetazolamide on perceived visual disability in every domain of the Michigan Retinal Degeneration Questionnaire.
**Table S9:** Effect of acetazolamide on perceived visual disability measured by Michigan Retinal Degeneration Questionnaire across every visit.
**Table S10:** Associations between structural and functional endpoints using mixed‐effects linear regression models accounting for within‐patient longitudinal change.
**Table S11:** Abnormal fundus imaging and fundus autofluorescence findings in XLRS patient per arm.
**Table S12:** Fundus autofluorescence findings in X‐linked retinoschisis patients per treatment arm.

## Data Availability

The data that support the findings of this study are available on request from the corresponding author. The data are not publicly available due to privacy or ethical restrictions.
