# Supplementary material for: Randomised Clinical Trial to Evaluate the Efficacy of Acetazolamide for the Treatment of Cystoid Fluid Collections in X‐Linked Retinoschisis: The AXIS Trial
Source: Clin Exp Ophthalmol. 2026 Aug 11;54(7):932–42. doi: 10.1111/ceo.70146 (PMC13643680; doi:10.1111/ceo.70146)
Supplement: Supplementary file 1 [file CEO-54-932-s002.pdf]

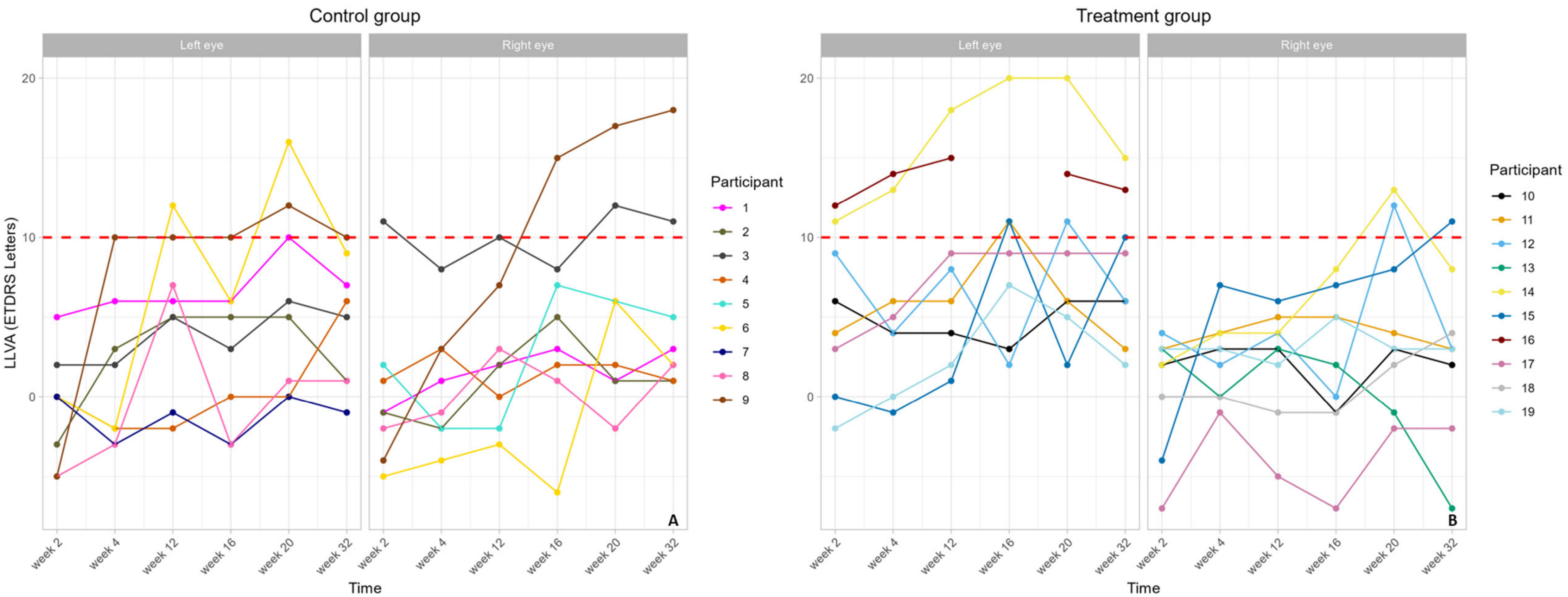


**Figure S1**: Changes in low luminance visual acuity (LLVA), measured in Early Treatment Diabetic Retinopathy Study (ETDRS) letters, from baseline to follow-up visits (week 2, 4, 12, 16, 20, and 32) in the control and treatment groups. Individual eye trajectories are shown separately for left and right eyes. The red dashed line represents a +10 ETDRS letter gain, denoting the threshold for clinically meaningful improvement. **A**, control group. **B**, treatment group.

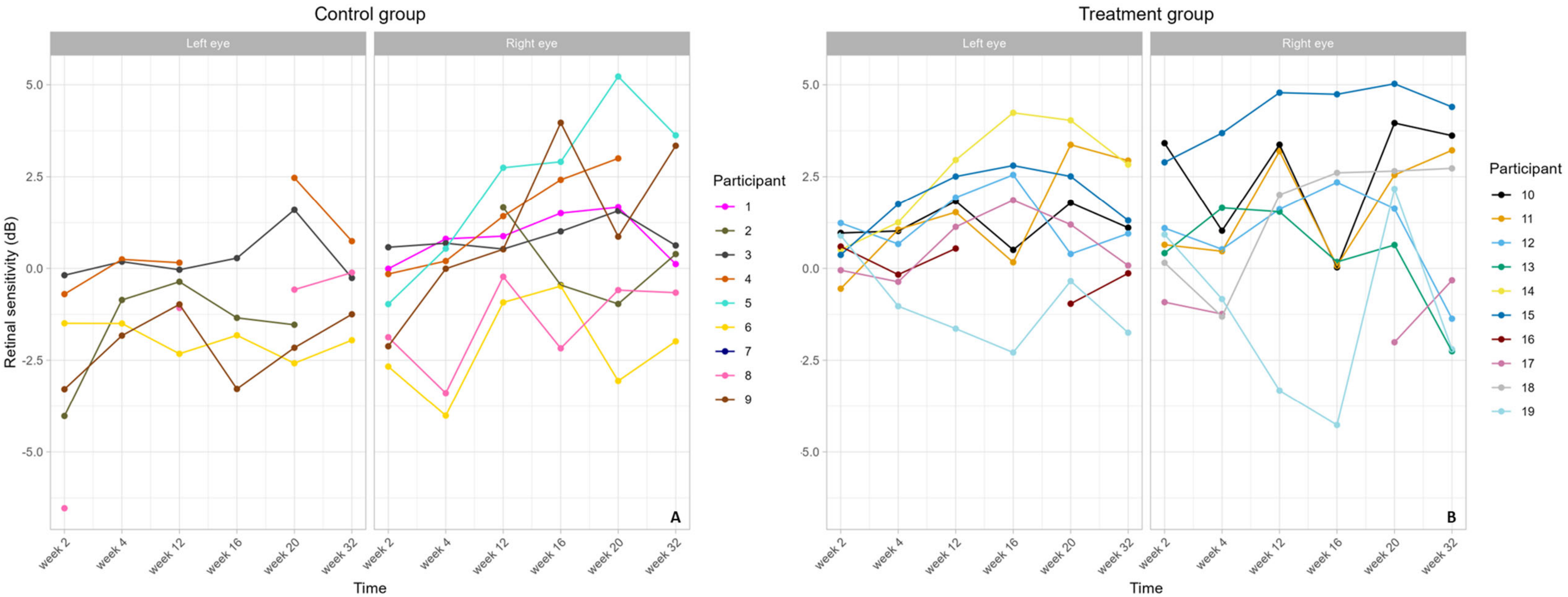


**Figure S2**: Changes in retinal sensitivity on microperimetry (MP, in dB) from baseline to follow-up visits (week 2, 4, 12, 16, 20, and 32) in the control and treatment groups. Individual eye trajectories are shown separately for left and right eyes. **A**, control group. **B**, treatment group.

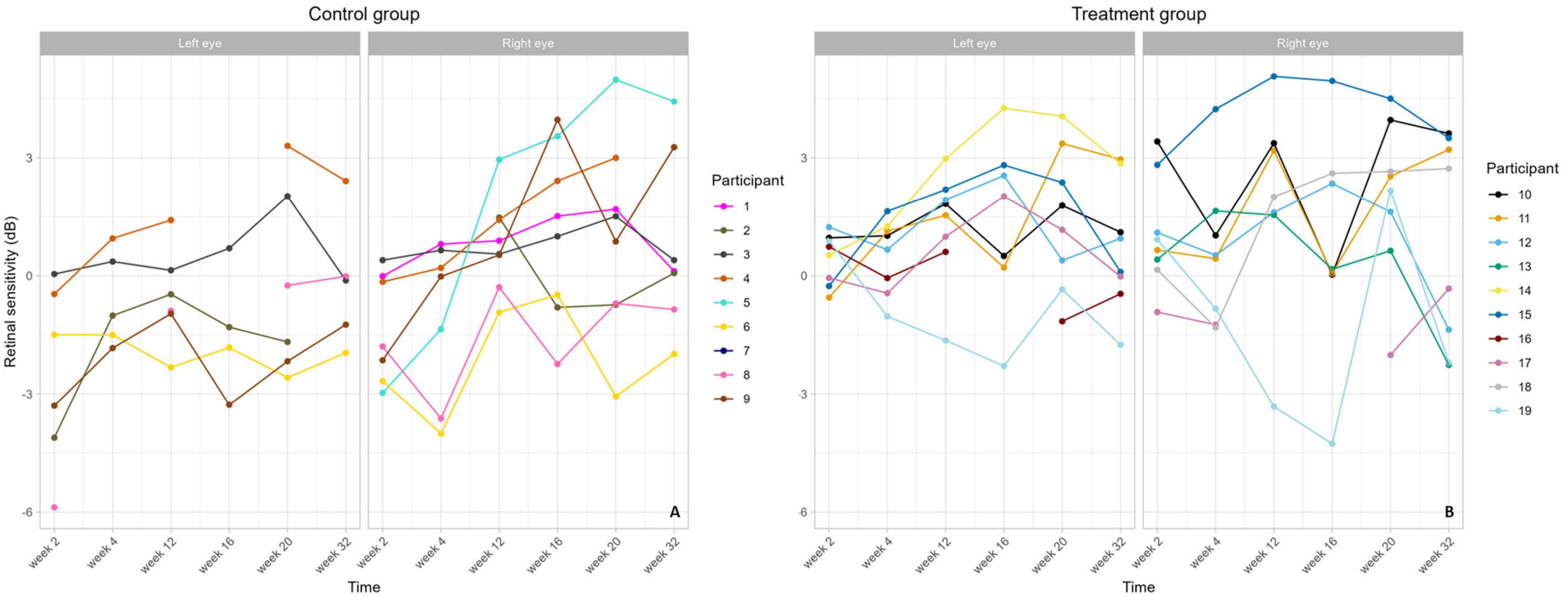


**Figure S3:** Changes in retinal sensitivity on microperimetry (MP, in dB) with the tested area restricted to cystoid fluid collections (CFC) area from baseline to follow-up visits (week 2, 4, 12, 16, 20, and 32) in the control and treatment groups. Individual eye trajectories are shown separately for left and right eyes. **A**, control group. **B**, treatment group.

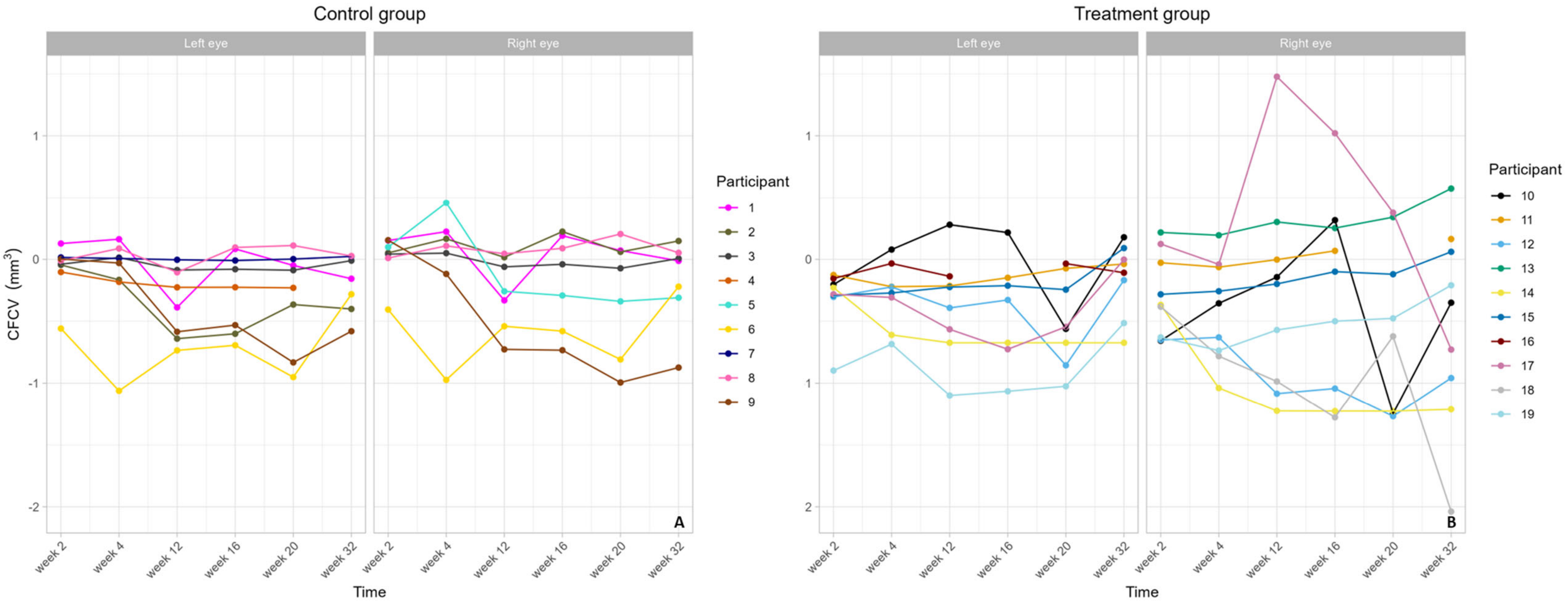


**Figure S4:** Changes in cystoid fluid collections volume (CFCV, in $mm^3$) from baseline to follow-up visits (week 2, 4, 12, 16, 20, and 32) in the control and treatment groups. Individual eye trajectories are shown separately for left and right eyes. **A**, control group. **B**, treatment group.
