# Supplementary material for: Randomised Clinical Trial to Evaluate the Efficacy of Acetazolamide for the Treatment of Cystoid Fluid Collections in X‐Linked Retinoschisis: The AXIS Trial
Source: Clin Exp Ophthalmol. 2026 Aug 11;54(7):932–42. doi: 10.1111/ceo.70146 (PMC13643680; doi:10.1111/ceo.70146)
Supplement: Supplementary file 2 [file CEO-54-932-s001.docx]

Supplementary Table S1: Genetic information on the pathogenic *Retinoschisin-1* variant in the 19 subjects with X-linked retinoschisis.

| Subject ID | Group allocation | Variant | Variant classification |
| --- | --- | --- | --- |
| 1 | Control | c.438G>C p.(Glu146Asp) | Missense |
| 2 | Control | N/A | N/A |
| 3 | Control | deletion exon 3 | Deletion |
| 4 | Control | N/A | N/A |
| 5 | Control | c.637C>T p.(Arg213Trp) | Missense |
| 6 | Control | c.214G>A p.(Glu72Lys) | Missense |
| 7 | Control | N/A | N/A |
| 8 | Control | c.412A>G p.(Thr138Ala) | Missense |
| 9 | Control | N/A | N/A |
| 10 | Treatment | c.214G>A p.(Glu72Lys) | Missense |
| 11 | Treatment | deletion exon 3 | Deletion |
| 12 | Treatment | c.214G>A p.(Glu72Lys) | Missense |
| 13 | Treatment | c.655delT p.(Cys219Alafs*18) | Frameshift |
| 14 | Treatment | c.271G>Tp.(Gly91Cys) | Missense |
| 15 | Treatment | deletion Exon 3 | Deletion |
| 16 | Treatment | c.214G>A p.(Glu72Lys) | Missense |
| 17 | Treatment | N/A | N/A |
| 18 | Treatment | c.214G>A p.(Glu72Lys) | Missense |
| 19 | Treatment | N/A | N/A |

Supplementary Table S2: Effect of acetazolamide on central subfield thickness across every visit.

| Effect of acetazolamide on central subfield thickness across every visit | | | |
| --- | --- | --- | --- |
| Visit | Coefficient (µm) | P-value | 95% confidence interval (µm) |
| Week 2 (visit 2) | -22.59 | 0.61 | -108.10 to 62.93 |
| Week 4 (visit 3) | -22.95 | 0.54 | -97.04 to 51.14 |
| Week 12 (visit 4) | +58.13 | 0.12 | -15.96 to 132.22 |
| Week 16 (visit 5) | +11.94 | 0.75 | -62.80 to 86.68 |
| Week 20 (visit 6) | +43.10 | 0.25 | -31.00 to 117.19 |
| Week 32 (visit 7) | +59.80 | 0.11 | -14.29 to 133.89 |

Supplementary Table S3: Effect of acetazolamide on best-corrected visual acuity across every visit.

| Effect of acetazolamide on best-corrected visual acuity across every visit | | | |
| --- | --- | --- | --- |
| Visit | Coefficient (ETDRS letters) | P-value | 95% confidence interval (ETDRS letters) |
| Week 2 (visit 2) | +0.85 | 0.52 | -1.73 to 3.43 |
| Week 4 (visit 3) | -0.14 | 0.90 | -2.37 to 2.09 |
| Week 12 (visit 4) | -0.37 | 0.74 | -2.60 to 1.86 |
| Week 16 (visit 5) | -0.28 | 0.81 | -2.53 to 1.97 |
| Week 20 (visit 6) | +0.61 | 0.59 | -1.62 to 2.84 |
| Week 32 (visit 7) | +0.48 | 0.67 | -1.75 to 2.71 |

Supplementary Table S4: Effect of acetazolamide on low luminance visual acuity across every visit.

| Effect of acetazolamide on low luminance visual acuity across every visit | | | |
| --- | --- | --- | --- |
| Visit | Coefficient (ETDRS letters) | P-value | 95% confidence interval (ETDRS letters) |
| Week 2 (visit 2) | +3.42 | 0.09 | -0.50 to 7.35 |
| Week 4 (visit 3) | -0.32 | 0.87 | -3.95 to 3.32 |
| Week 12 (visit 4) | -2.07 | 0.27 | -5.70 to 1.57 |
| Week 16 (visit 5) | -1.37 | 0.46 | -5.04 to 2.30 |
| Week 20 (visit 6) | -2.24 | 0.23 | -5.88 to 1.39 |
| Week 32 (visit 7) | -3.02 | 0.10 | -6.66 to 0.61 |

Supplementary Table S5: Effect of acetazolamide on retinal sensitivity on total tested area on microperimetry across every visit.

| Effect of acetazolamide retinal sensitivity on total tested area on microperimetry across every visit | | | |
| --- | --- | --- | --- |
| Visit | Coefficient (dB) | P-value | 95% confidence interval (dB) |
| Week 2 (visit 2) | +2.59 | 0.001 | 1.08 to 4.11 |
| Week 4 (visit 3) | -1.20 | 0.08 | -2.53 to 0.12 |
| Week 12 (visit 4) | -1.23 | 0.07 | -2.54 to 0.08 |
| Week 16 (visit 5) | -1.82 | 0.01 | -3.17 to -0.46 |
| Week 20 (visit 6) | -1.19 | 0.07 | -2.49 to 0.11 |
| Week 32 (visit 7) | -1.99 | 0.003 | -3.32 to -0.66 |

Supplementary Table S6: Effect of acetazolamide on retinal sensitivity on tested area on microperimetry restricted to cystoid fluid collections area across every visit.

| Effect of acetazolamide on tested area on microperimetry restricted to cystoid fluid collections-area across every visit | | | |
| --- | --- | --- | --- |
| Visit | Coefficient (dB) | P-value | 95% confidence interval (dB) |
| Week 2 (visit 2) | +2.75 | <0.001 | +1.24 to 4.25 |
| Week 4 (visit 3) | -1.11 | 0.12 | -2.50 to 0.28 |
| Week 12 (visit 4) | -1.42 | 0.04 | -2.80 to -0.05 |
| Week 16 (visit 5) | -1.99 | 0.01 | -3.42 to -0.57 |
| Week 20 (visit 6) | -1.41 | 0.04 | -2.78 to -0.05 |
| Week 32 (visit 7) | -2.39 | 0.001 | -3.78 to -0.99 |

Supplementary Table S7: Effect of acetazolamide on cystoid fluid collections volume across every visit.

| Effect of acetazolamide on cystoid fluid collections volume across every visit | | | |
| --- | --- | --- | --- |
| Visit | Coefficient (mm^3^) | P-value | 95% confidence interval (mm^3^) |
| Week 2 (visit 2) | -0.08 | 0.58 | -0.37 to 0.21 |
| Week 4 (visit 3) | -0.00 | 1.00 | -0.30 to 0.30 |
| Week 12 (visit 4) | +0.26 | 0.10 | -0.05 to 0.56 |
| Week 16 (visit 5) | +0.15 | 0.32 | -0.15 to 0.46 |
| Week 20 (visit 6) | +0.06 | 0.72 | -0.25 to 0.36 |
| Week 32 (visit 7) | +0.10 | 0.52 | -0.21 to 0.41 |

Supplementary Table S8: Effect of acetazolamide on perceived visual disability in every domain of the Michigan Retinal Degeneration Questionnaire.

| MRDQ-domain | Coefficient (θ) | P-value | 95% confidence interval (θ) |
| --- | --- | --- | --- |
| Central vision | -0.05 | 0.66 | -0.62 to 0.16 |
| Colour vision | -0.15 | 0.31 | -0.40 to 0.09 |
| Contrast | -0.12 | 0.23 | -0.34 to 0.11 |
| Scotopic | -0.15 | 0.25 | -0.41 to 0.11 |
| Photopic peripheral | +0.14 | 0.17 | -0.06 to 0.36 |
| Mesopic peripheral | -0.06 | 0.61 | -0.29 to 0.17 |
| Photosensitivity | -0.15 | 0.20 | -0.38 to 0.08 |

Supplementary Table S9: Effect of acetazolamide on perceived visual disability measured by Michigan Retinal Degeneration Questionnaire across every visit.

| Effect of acetazolamide on perceived visual disability measured by Michigan Retinal Degeneration Questionnaire across every visit | | | | |
| --- | --- | --- | --- | --- |
| Domain | Visit | Coefficient (θ) | P-value | 95% confidence interval (θ) |
| Central vision | Week 12 (visit 4) | +0.01 | 0.93 | -0.22 to 0.24 |
|  | Week 16 (visit 5) | +0.01 | 0.87 | -0.17 to 0.19 |
|  | Week 32 (visit 7) | -0.18 | 0.05 | -0.36 to -0.004 |
| Colour vision | Week 12 (visit 4) | -0.21 | 0.13 | -0.47 to 0.06 |
|  | Week 16 (visit 5) | +0.12 | 0.21 | -0.07 to 0.30 |
|  | Week 32 (visit 7) | +0.05 | 0.55 | -0.12 to 0.23 |
| Contrast | Week 12 (visit 4) | -0.10 | 0.42 | -0.34 to 0.14 |
|  | Week 16 (visit 5) | +0.06 | 0.38 | -0.08 to 0.20 |
|  | Week 32 (visit 7) | -0.11 | 0.12 | -0.25 to 0.03 |
| Scotopic | Week 12 (visit 4) | -0.08 | 0.55 | -0.36 to 0.19 |
|  | Week 16 (visit 5) | -0.11 | 0.19 | -0.26 to 0.05 |
|  | Week 32 (visit 7) | -0.11 | 0.17 | -0.26 to 0.05 |
| Photopic peripheral | Week 12 (visit 4) | +0.20 | 0.09 | -0.04 to 0.45 |
|  | Week 16 (visit 5) | -0.11 | 0.31 | -0.32 to 0.11 |
|  | Week 32 (visit 7) | -0.08 | 0.47 | -0.28 to 0.12 |
| Mesopic peripheral | Week 12 (visit 4) | +0.03 | 0.76 | -0.22 to 0.30 |
|  | Week 16 (visit 5) | -0.11 | 0.23 | -0.30 to 0.07 |
|  | Week 32 (visit 7) | -0.19 | 0.04 | -0.37 to -0.01 |
| Photosensitivity | Week 12 (visit 4) | -0.04 | 0.75 | -0.30 to 0.22 |
|  | Week 16 (visit 5) | -0.11 | 0.37 | -0.34 to 0.13 |
|  | Week 32 (visit 7) | -0.20 | 0.09 | -0.43 to 0.03 |

**Supplementary** **Table S10: Associations between structural and functional endpoints using mixed-effects linear regression models accounting for within-patient longitudinal change**

|  | Change in CST | | | | | | Change in CFCV | | | | | |
| --- | --- | --- | --- | --- | --- | --- | --- | --- | --- | --- | --- | --- |
|  | Coefficient (µm) | Z-score | P-value | 95% confidence interval (µm) | Number of observations | Numberof groups | Coefficient (mm^3^) | Z-score | P-value | 95% confidence interval (mm^3^) | Number of observations | Number of groups |
| Change in BCVA (ETDRS letters) | -0.0092 | -4.42 | <0.001 | -0.013 to -0.0051 | 197 | 19 | -2.40 | -4.33 | <0.001 | -3.48 to -1.31 | 189 | 19 |
| Change in LLVA (ETDRS letters) | -0.012 | -3.63 | <0.001* | -0.019 to -0.0056 | 197 | 19 | -3.58 | -4.03 | <0.001* | -5.32 to -1.84 | 189 | 19 |
| Change in retinal sensitivity on total tested MP area (dB) | -0.0050 | -3.86 | <0.001* | -0.01 to -0.002 | 170 | 18 | -1.29 | -3.49 | <0.001* | -2.02 to -0.57 | 163 | 18 |
| Change in retinal sensitivity on MP restricted to CFC-area (dB) | -0.0063 | -4.66 | <0.001* | -0.0089 to -0.0036 | 170 | 18 | -1.46 | -3.77 | <0.001* | -2.22 to -0.70 | 163 | 18 |

* Coefficients reflect associations adjusted for treatment allocation (acetazolamide vs. control), eye (right vs. left), and repeated measures within patients using mixed-effects models with a random intercept. CST coefficients are rounded to two decimal places for clarity. Treatment was a significant covariate in CST models for change in retinal sensitivity on the total MP-tested area (p = 0.021) and MP restricted to the CFC area (p = 0.027). Eye laterality was significant in CST models for LLVA (p = 0.001), total MP area (p < 0.001), and CFC-restricted MP (p = 0.001), and in CFCV models for LLVA (p < 0.001), total MP area (p = 0.003), and CFC-restricted MP (p = 0.007).

Supplementary Table S11: Abnormal fundus imaging and fundus autofluorescence findings in XLRS patient per arm.

|  | Number of subjects (eyes)  Treatment | Number of subjects (eyes)  Control | Number of subjects (eyes)  Treatment | Number of subjects (eyes)  Control |
| --- | --- | --- | --- | --- |
| Category | At baseline visit  (N=10, 17) | At baseline visit  (N=9, 16) | At final visit  (N=10, 17) | At final visit  (N=9, 16) |
| Optic disc |  |  |  |  |
| Dragged | 1 (1) | 1 (1) | 1 (1) | 1 (1) |
| Peripapillary atrophy | - | 2 (2) | - | 2 (2) |
| Rim thinning | 1 (1) | - | 1(1) | - |
| Macula |  |  |  |  |
| Retinoschisis | 4 (8) | 4 (6) | 3 (6) | 3 (5) |
| Spoke wheel pattern | - | 2 (2) | 1 (2) | 1 (1) |
| Peripheral retina |  |  |  |  |
| Peripheral schisis | 5 (7) | 3 (3) | 5 (7) | 3 (3) |
| Lattice-like dendritic changes | 2 (3) | 3 (3) | 2 (3) | 3 (3) |
| Golden sheen | 7 (12) | 4 (5) | 7 (12) | 4 (5) |
| Bear tracks | - | 1 (2) | - | 1 (2) |
| Blonde fundus | 1 (2) | - | 1 (2) | - |
| Retinal vasculature |  |  |  |  |
| Vascular sheeting | 1 (1) | - | 1 (1) | - |
| Vascular tortuosity | 1 (1) | - | 1 (1) | - |
| Vitreous |  |  |  |  |
| Floaters | - | 2 (3) | - | 2 (3) |
| Vitreous veils | 1 (1) | - | 1 (1) | - |
| Vitreous strands | 1 (1) | - | 1 (1) | - |

Supplementary Table S12: Fundus autofluorescence findings in X-linked retinoschisis patients per treatment arm.

|  | Number of subjects (eyes)  Treatment | Number of subjects (eyes)  Control | Number of subjects (eyes)  Treatment | Number of subjects (eyes)  Control |
| --- | --- | --- | --- | --- |
| Category | At baseline visit  (N=10, 17) | At baseline visit  (N=9, 16) | At final visit  (N=10, 17) | At final visit  (N=9, 16) |
| Fovea hyper-autofluorescence | 5 (8) | 3 (5) | 5 (7) | 3 (5) |
| Hyperfluorescent macular ring | - | 2 (4) | - | 2 (4) |
| Spoke wheel pattern | 9 (14) | 8 (14) | 8 (12) | 7 (13) |
