# Supplementary material for: Randomised Clinical Trial to Evaluate the Efficacy of Acetazolamide for the Treatment of Cystoid Fluid Collections in X‐Linked Retinoschisis: The AXIS Trial
Source: Clin Exp Ophthalmol. 2026 Aug 11;54(7):932–42. doi: 10.1111/ceo.70146 (PMC13643680; doi:10.1111/ceo.70146)
Supplement: Supplementary file 3 [file CEO-54-932-s003.docx]

**Supplementary Methods protocol**

*Study design*

The AXIS trial was an investigator-initiated, single centre, open-label, randomised controlled trial consisting of seven visits conducted at the Amsterdam University Medical Centre (Amsterdam, the Netherlands). This prospective study adhered to the tenets of the Declaration of Helsinki and was approved by the institutional review board and ethics committee of Amsterdam University Medical Centre in Amsterdam (NL80249.018.22). The AXIS trial (Randomised Clinical Trial to Evaluate the Efficacy of Acetazolamide for the Treatment of Cystoid Fluid Collections in Retinoschisis) was registered at ClinicalTrials.gov (identifier, NCT06114537) and assigned the EudraCT number 2002-000237-17. We obtained written informed consent from all enrolled patients prior to their participation.

*Participants*

Male patients aged 12 years or older, with a molecularly confirmed diagnosis (pathogenic variants in *RS1*) of XLRS, were recruited for this study. Patients could also be enrolled without genetic testing based on the following criteria: an X-linked disease inheritance pattern; decreased visual acuity; and typical central retinoschisis on SD-OCT. In addition to being eligible for inclusion, the presence of CFC involving the fovea on SD-OCT in at least one eye was required at the baseline visit. Exclusion criteria in this trial were visual dysfunction associated with amblyopia or another ocular disease other than XLRS; BCVA of 20/200 or worse (Snellen equivalent); use of any CAI within the last 3 months; use of medications that interact with acetazolamide (see Table 1) or any contraindications to receiving acetazolamide or mydriatics (tropicamide 0.5% or phenylephrine 5%). Patients previously on CAI treatment could be eligible after a washout period of three months. The complete list of inclusion and exclusion criteria is found in Table 1.

*Randomisation*

Patients were randomly assigned (at a ratio of 1:1) to receive either acetazolamide treatment or active observation. The randomisation was performed using a web-based random number generator with block randomisation using block sizes of 5. Participants or investigators could not be masked to treatment assignment due to the absence of a placebo.

*Procedures*

The demographics, medical history, and concurrent medication use of each patient were recorded at the baseline visit. The trial consisted of seven visits at 2, 4, 12, 16, 20, and 32 weeks following the baseline evaluation visit. Each patient was scheduled at the same time of day for each appointment to account for diurnal fluctuation in visual function and OCT morphology, which has been previously described in XLRS.^1^ The following measurements were obtained at baseline and all follow-up visits: BCVA in Early Treatment of Diabetic Retinopathy Study (ETDRS) letters; low luminance visual acuity (LLVA) in ETDRS letters; retinal sensitivity on microperimetry using a Macular Integrity Assessment microperimeter (MAIA; iCare, Padova, Italy); OCT scanning of the macula. We assessed the functional (dis)ability at baseline, 4^th^ (week 12), 5^th^ (week 16), and final visit (week 32) using the Michigan Retinal Degeneration Questionnaire (MRDQ). Additionally, fundus autofluorescence (FAF) images and fundus imaging were collected at both the baseline and final visits. Multimodal imaging consisted of ultra-widefield retinal images of the fundus (200° image) acquired on the Optos California device (Optos PLC, Dunfermline, United Kingdom), and SD-OCT and FAF imaging acquired on the Heidelberg Spectralis HRA+OCT device (Heidelberg Engineering Inc., Heidelberg, Germany).

The BCVA and LLVA were measured without formal dark adaptation, using established protocols with the retroilluminated ETDRS letter charts (Precision Vision, Bloomington, IL, USA). The LLVA was measured by adding a 2-log neutral density filter. Retinal sensitivity was measured using microperimetry in undilated eyes under mesopic conditions without formal dark adaptation. We used the expert 4-2 strategy and a standard 10°-grid with 37 stimuli targeted at the preferred retinal location (PRL) to accomodate an eccentric fixation. To minimize learning effects in microperimetry testing, participants completed a practice session during the first three visits (fast, supra-threshold test mode) before undertaking the formal test (expert, 4–2 strategy). Fixation stability and reliability were evaluated using the 95% fixation bivariate contour ellipse area (BCEA95), which encompasses 95% of fixation points within the standard deviations of the horizontal and vertical eye positions during the fixation attempt. Microperimetry testing was considered reliable if fixation loss was ≤30% and the BCEA95 <50 deg².^2,3^

The MRDQ contains 59 Likert-scaled questions, with item response theory analysis generating a theta score (θ) as a metric for person-level (dis)ability in a specific domain or trait.^4^ The original authors granted permission to use the MRDQ for research purposes, and an end-user license agreement was signed by all parties involved. For Dutch-speaking participants, we used the Dutch version of the MRDQ, which was translated from English via a forward–backward translation process by an external translation agency, as provided in the supplementary materials of the study by Karuntu et al.^5^ All clinicians and researchers involved in collecting the data were trained and certified, and all data were obtained using standard operating procedures.

*Acetazolamide treatment*

Patients in the treatment group started at 500 mg/day for 16 weeks from the baseline visit. The tablets were taken twice a day (250 mg bid) orally with no restriction on food consumption. Sufficient tablets were dispensed at each visit to last for at least to the next follow-up visit. Patients were asked to return all remaining acetazolamide tablets at each visit, allowing the calculation of the difference between the expected number and the actual number of tablets taken.

At week 16, a treatment decision point was introduced based on the degree of CFC reduction (Figure 1), with a decrease of at least 25% in central foveal thickness (CFT) considered clinically significant, following a cut-off value of 22.4% decrease in CFT in previous studies.^6,7^ The CFT was selected over central subfield thickness (CST) at this time point due to time constraints during study visits, which precluded manual correction of the automated retinal layer segmentation errors by the in-built software (Spectralis software version 6.0; Heidelberg Engineering, Heidelberg, Germany). In our prior work, CFT and CST demonstrated a strong correlation (Spearman’s ρ = 0.97), supporting the use of CFT as a reliable proxy in this context.^8^

The treatment was discontinued for patients without a reduction in CFT of ≥25% in either of the study eyes and they proceeded with observation only. Patients showing a reduction of ≥25% or complete CFC resolution in either study eye, transitioned to a reduced dose of 125 mg twice daily for four weeks, until the next visit at week 20. Following these four weeks at a reduced dose, the CFT was re-evaluated. If CFT increased and fell below a 25% reduction from baseline, the dosage was adjusted back to 250 mg twice daily until the final visit at week 32. Patients with a complete resolution of CFC, further decrease in CFT or relatively stable CFT who maintained a reduction of ≥25% in at least one study eye, continued with the reduced dose of 125 mg twice daily until the end of the study, if side effects remained tolerable and no interim dose adjustments were required (Figure 1). A side effect questionnaire was used at each visit to assess drug tolerability. If intolerable side effects occurred at the 250 mg twice-daily dose, we attempted to alleviate the side effects by providing dietary advice (food products containing high potassium). In case of persisting adverse events, the dosage was either reduced to 125 mg twice daily or discontinued, as agreed with the patient.

*Primary and secondary endpoints*

The primary outcome was the difference in CST on SD-OCT over the 32-week study period on average over time between the treatment and control groups. Secondary outcomes included the difference in ETDRS BCVA, ETDRS LLVA, mean retinal sensitivity on microperimetry (both for the total tested field and restricted to CFC area), the cystoid fluid collections volume (CFCV) on SD-OCT, and vision-related quality of life using MRDQ. Adverse events and serious adverse events were monitored and reported to the data safety monitoring board, which had the authority to terminate the study early if deemed necessary.

Retinal sensitivity on microperimetry data

To accurately quantify retinal function, we generated smoothed and interpolated sensitivity maps using the "hill of vision" concept, which estimates the total area of sensitivity across the tested visual field (Figure 2A-B). This two-dimensional interpolation method provides an unbiased estimate of sensitivity across the entire tested area. In contrast, native mean sensitivity inherently biases results toward the grid's centre due to the radial concentration of test points in the 37-point layout.^9^ Interpolated sensitivity maps were created using publicly available R-code by Josan et al.^9^

To maintain consistency in analyses across multiple visits, all interpolated sensitivity maps were aligned based on vessel bifurcations and loaded into FIJI ImageJ as an image stack. A common retinal area was defined by creating circular regions of interest (ROIs) for each sensitivity map and calculating the intersection of these regions. This overlapping ROI served as a standardised region to crop each map, correcting for positional shifts of the grid across follow-up assessments.

As an additional outcome measure, microperimetry sensitivity was specifically analysed within regions exhibiting CFC identified at baseline. We used our previously validated artificial intelligence (AI) algorithm to overlay CFC segmentations onto Spectralis scanning laser ophthalmoscopy images.^10^ These overlays were manually aligned with the interpolated sensitivity maps through identifiable anatomical landmarks (Figure 2C-F). Subsequently, the CFC-specific regions of interest were cropped consistently to the shared retinal area for all visits, facilitating precise evaluation of functional changes within CFC-affected retinal regions.

Cystoid fluid collections volume (CFCV)

The CFCV was quantified from the SD-OCT volume scans using another recently developed and validated artificial intelligence-based automated segmentation model specifically designed for volume OCT-scans of XLRS patients.^11^ This AI model is able to provide automated segmentation of CFC in individual raster B-scans, and quantify CFCV measurements in whole volume scans, to track longitudinal changes over time. These AI-based CFCV measurements have been validated to be comparable to gold standard human annotation.^11^ In this study, to further ensure data reliability, an additional quality control pipeline was implemented. First, we applied an automated outlier detection method at the individual raster B-scan level, using a Savitzky–Golay smoothing filter to estimate expected values for each B-scan based on local neighbouring B-scans. Individual B-scans with ≥35% deviation from their expected values were flagged as potential outliers, provided the measured fluid area was at least 500 pixels (to avoid noise at low values). The filtering parameters included a window length of 5 slices and a polynomial order of 3. Additionally, a customised edge-mirroring strategy was used to improve detection near scan borders, where neighbouring data may be sparse. All scans flagged as potential outliers underwent manual review by a trained grader (J.H.). Scans were retained if flagged slices were deemed correctly segmented; scans with one or more slices containing confirmed segmentation errors were excluded from analysis.

Michigan Retinal Degeneration Questionnaire (MRDQ).

Patient responses on the MRDQ were evaluated using the Graded Response Model, employing Cai's Metropolis-Hastings Robbins-Monro algorithm from the mirt package in R, as previously described by the original authors.^4^ For each MRDQ domain, θ-scores (theta) were calculated to reflect the patient’s level of visual disability, ranging from −3 to +3. Higher θ-scores indicate greater difficulty or limitation in the respective domain. In this analysis, θ is centred on the mean trait level of the original study population, which included 128 patients with inherited retinal dystrophies (69 rod-cone (53.9%), 30 cone/cone-rod (23.4%), and 29 macular dystrophies (22.7%).^4^ Extreme scores represent low (−3) or high (+3) levels of perceived visual dysfunction based on item responses.

*Statistical analyses*

A target sample size of 18 patients was calculated to detect a clinically relevant treatment effect, defined as a 25% reduction in CST at week 32, assuming a standard deviation of 90 µm, with 80% power at a two-sided α level of 0.05 and allowing for a 10% dropout rate. This threshold was based on prior studies in XLRS that considered a ≥22.4% reduction in CST to be clinically relevant.^6,7^ The calculation was originally based on one eye per participant. However, both eyes were included in the final analysis when eligible, which increased the effective sample size. To account for within-subject and between-eye correlation, a correlation coefficient of 0.6 was assumed. Each participant underwent seven visits over a 32-week period (baseline visit plus six follow-up visits). All analyses followed the intention-to-treat principle. Continuous variables are presented as mean ± standard deviation (SD) or median with interquartile range (IQR), depending on distribution. Categorical variables are reported as frequencies and percentages. Mixed-effects linear regression models were used to evaluate treatment effect for continuous longitudinal outcomes. For outcomes assessed at baseline and follow-up visits, baseline measurements were included as covariates. Two longitudinal analyses were performed. The first only included treatment, which resulted in a treatment effect on average over time, while the second included treatment, time, and the time-by-treatment interaction, which resulted in treatment effects at each follow-up visit. Furthermore, all analyses were adjusted for laterality (eye) and a random intercept on patient level was added to the model to account for the intra-subject correlation. Associations between changes in structural (CST and CFCV) and functional outcomes (BCVA, LLVA, and retinal sensitivity (restricted to CFC area)) were also analysed using mixed-effects linear regression models. These models included the changes between subsequent measurements and the analyses were adjusted for treatment group and laterality (eye), and included a random intercept on patient level to account for the intra-subject correlation.

All statistical analyses were conducted using SPSS Statistics (version 25.0; IBM Corp., Armonk, NY, USA), except for the calculations of the θ-scores for each MRDQ domain using R software version 4.4.3 (R Foundation for Statistical Computing, Vienna, Austria), and the mixed-effects linear regression models, which were performed in Stata (version 15.1; StataCorp, College Station, TX, USA).

Table 1 Inclusion and exclusion criteria of the AXIS trial.

Inclusion Criteria

≥12 years of age and able to give written informed consent

Confirmed diagnosis of XLRS

Foveal CFC on SD-OCT at the baseline examination

Exclusion Criteria

Presence of amblyopia or any other ocular diseases (e.g., glaucoma, perforating trauma) that could contribute to visual dysfunction, aside from XLRS

BCVA of 20/200 or worse (Snellen equivalent)

Concurrent treatment with CAI (topical or systemic), use of any CAI within three months before the expected start of the trial

Intolerance or allergy to ocular anaesthetic eye drops (oxybuprocaine 0.4%) or mydriatics (tropicamide 0.5%, phenylephrine 5%)

Contraindications to receiving acetazolamide

- Severe hepatic impairment
- Severe renal insufficiency
- Sodium or potassium depletion
- Addison’s disease
- Hyperchloremic acidosis
- Cor pulmonale
- Chronic non-congestive angle-closure glaucoma

Use of medications that interact with acetazolamide

- Folic acid antagonists (methotrexate, trimethoprim, pyrimethamine)
- Oral anticoagulants
- Hypoglycaemic agents (metformin)
- Aspirin
- Cardiac glycosides (digoxin)
- Diuretics (e.g., thiazides, loop diuretics)
- Anticonvulsants (phenytoin, primidone, carbamazepine)
- Carbonic anhydrase inhibitors (methazolamide, dorzolamide, brinzolamide, diclofenamide, ethoxzolamide, and zonisamide)
- Procaine
- Sodium hydrogen carbonate
- Cyclosporine
- Ephedrine, methadone, amphetamine, quinidine, lithium

BCVA = best-corrected visual acuity; CAI = carbonic anhydrase inhibitor; CFC = cystoid fluid collections; OCT = optical coherence tomography; SD = spectral-domain; XLRS = X-linked retinoschisis.

**Figure 1.** Flowchart of acetazolamide treatment protocol. CFC = cystoid fluid collections; CFT = central foveal thickness;

**Figure 2**: Steps required to measure retinal sensitivity on microperimetry when the tested area is restricted to area containing cystoid fluid collections (CFC). (**A**,) An example of the standard Point threshold values overlaid onto a fundus image output given by the MAIA microperimeter. (**B**,) Two dimensional smoothed, interpolated heatmap generated using thin plate spline. (**D**,) An example of the standard Spectralis scanning laser ophthalmoscopy (SLO) image of the same patient. (**E**,) Heatmap of CFC predictions overlaid onto the Spectralis SLO-image. (**F**,) Manually aligned and transformed the SLO-image to the interpolated sensitivity map by identifying anatomical landmarks. (**C**,) Two dimensional smoothed, interpolated heatmap of the point sensitivities restricted to CFC-area.

**References**

1.	Mautone L, Atiskova Y, Druchkiv V, Spitzer MS, Dulz S. Diurnal functional and anatomical changes in X-linked retinoschisis. *Graefes Arch Clin Exp Ophthalmol*. Nov 2023;261(11):3307–3313. doi:10.1007/s00417-023-06106-0

2.	Josan AS, Farrance I, Taylor LJ, et al. Microperimetry Reliability Assessed From Fixation Performance. *Transl Vis Sci Technol*. May 1 2023;12(5):21. doi:10.1167/tvst.12.5.21

3.	Higgins BE, Montesano G, Dunbar HMP, et al. Test-Retest Variability and Discriminatory Power of Measurements From Microperimetry and Dark Adaptation Assessment in People With Intermediate Age-Related Macular Degeneration - A MACUSTAR Study Report. *Transl Vis Sci Technol*. Jul 3 2023;12(7):19. doi:10.1167/tvst.12.7.19

4.	Lacy GD, Abalem MF, Andrews CA, et al. The Michigan Retinal Degeneration Questionnaire: A Patient-Reported Outcome Instrument for Inherited Retinal Degenerations. *Am J Ophthalmol*. Feb 2021;222:60–68. doi:10.1016/j.ajo.2020.08.032

5.	Karuntu JS, Nguyen XT, Boon CJF. Correlations between the Michigan Retinal Degeneration Questionnaire and visual function parameters in patients with retinitis pigmentosa. *Acta Ophthalmol*. Aug 2024;102(5):555–563. doi:10.1111/aos.16601

6.	Verbakel SK, van de Ven JP, Le Blanc LM, et al. Carbonic Anhydrase Inhibitors for the Treatment of Cystic Macular Lesions in Children With X-Linked Juvenile Retinoschisis. *Invest Ophthalmol Vis Sci*. Oct 1 2016;57(13):5143–5147. doi:10.1167/iovs.16-20078

7.	Testa F, Di Iorio V, Gallo B, et al. Carbonic anhydrase inhibitors in patients with X-linked retinoschisis: effects on macular morphology and function. *Ophthalmic Genet*. Jun 2019;40(3):207–212. doi:10.1080/13816810.2019.1616303

8.	Hensman J, Hahn LC, van Schooneveld MJ, et al. Efficacy of Carbonic Anhydrase Inhibitors on Cystoid Fluid Collections and Visual Acuity in Patients with X-Linked Retinoschisis. *Ophthalmol Retina*. Jun 2024;8(6):600–606. doi:10.1016/j.oret.2023.12.003

9.	Josan AS, Buckley TMW, Wood LJ, Jolly JK, Cehajic-Kapetanovic J, MacLaren RE. Microperimetry Hill of Vision and Volumetric Measures of Retinal Sensitivity. *Transl Vis Sci Technol*. Jun 1 2021;10(7):12. doi:10.1167/tvst.10.7.12

10.	Hensman J, El Allali Y, Almushattat H, de Vente C, Sanchez CI, Boon CJF. Deep learning model for detecting cystoid fluid collections on optical coherence tomography in X-linked retinoschisis patients. *Acta Ophthalmol*. Apr 4 2025;doi:10.1111/aos.17495

11.	Tan TE, Dai P, Hensman J, et al. Artificial intelligence-quantified schisis volume as a structural endpoint for gene therapy clinical trials in X-linked retinoschisis. *Acta Ophthalmol*. Mar 29 2025;doi:10.1111/aos.17485
